# Toothbrushing, Blood Glucose and HbA1c: Findings from a Random Survey in Chinese Population

**DOI:** 10.1038/srep28824

**Published:** 2016-07-07

**Authors:** Lingyu Su, Wenzhao Liu, Bingwu Xie, Lei Dou, Jun Sun, Wenjuan Wan, Xiaoming Fu, Guangyue Li, Jiao Huang, Ling Xu

**Affiliations:** 1 College of Stomatology, Chongqing Medical University, Chongqing, China; 2 Chongqing Research Center for Oral Diseases and Biomedical Science, Chongqing, China; 3 Chongqing Municipal Key Laboratory of Oral Biomedical Engineering of Higher Education, Chongqing, China; 4 The First Affiliated Hospital of Chongqing Medical University, Chongqing Key Laboratory of Ophthalmology and Chongqing Eye Institute, Chongqing 400016, P.R. China

## Abstract

Both diabetes and periodontal disease are prevalent in China. Poor oral hygiene practice is the major cause of periodontal disease. An association between oral hygiene practice and blood glucose level was reported in individuals with diabetes, but not in the general population. We examined the association in a population-based random survey recruiting 2,105 adults without previously diagnosed diabetes in Chongqing city, China. Plasma glucose and hemoglobin A1c (HbA1c) were measured, and a 2-hour oral glucose tolerance test was conducted for each respondent. Self-reported toothbrushing frequency was used as a proxy for oral hygiene practice. In a linear model controlling for potential confounders (demographic characteristics, socio-economic status, lifestyle risk factors, BMI, dental visit frequency, etc.), urban residents who barely brushed their teeth had an increase of 0.50 (95% CI: 0.10–0.90) mmol/L in fasting plasma glucose, and an increase of 0.26% (0.04–0.47%) in HbA1c, relative to those brushing ≥twice daily; for rural residents, the effects were 0.26 (0.05–0.48) mmol/L in fasting plasma glucose and 0.20% (0.09–0.31%) in HbA1c. Individuals with better oral practice tended to have lower level of blood glucose and HbA1c. Establishing good oral health behavioral habits may be conducive to diabetes prevention and control in the general population.

Diabetes has become a major public health problem in China, increasing rapidly in last decades^1^,^2^. Periodontal disease, one of the most common chronic inflammatory diseases, may place individuals at increased risk of internal inflammation which may result in insulin resistance^3^,^4^. It was reported that over 85% Chinese adults suffered from periodontal disease^5^. There has been of great interest in the possible link between periodontal disease and diabetes or blood glucose control. A previous review considered the relationship between periodontitis and type 2 diabetes as bidirectional, with diabetes leading to increased risk for periodontitis, and periodontal inflammation negatively affecting glycemic control^6^. A number of studies revealed correlations of periodontal diseases with impaired fasting glucose, impaired glucose tolerance and HbA1c level^7^,^8^,^9^,^10^. Poor oral hygiene is the most important cause of periodontal disease. Self-reported measures of oral hygiene have been associated with periodontal disease^11^. The most predictable indicator of oral health practice is toothbrushing frequency, a measure which is commonly available in oral health-related surveys. A community based study analyzed data of routine health examination of 54,551 adults and reported that individuals who hardly brush teeth had higher odds of being diabetes (multivariate adjusted odds ratio: 1.61) than those who brush after each meal^12^. Several other studies revealed an association of toothbrushing behavior with HbA1c among diabetic patients^13^,^14^. For example, HbA1c control was found associated with toothbrushing ≥1 times daily (multivariate adjusted odds ratio: 3.1) in a cross-sectional study recruiting 155 participants <20 years with physician-diagnosed diabetes^12^; an Indian study reported that percentage of twice-daily toothbrushing was significantly higher in group of good diabetes control (Hba1c <6.0%) than in group of poor control (Hba1c > 7.5%) in both male and female adults with type 2 diabetes^14^. However, the association between oral hygiene behavior with level of blood glucose and HbA1c has not been previously examined in the general population. In this study, we investigated whether and to what extent toothbrushing frequency is associated with the level of blood glucose and HbA1c in China.

## Results

In general, a high percentage of the 2105 respondents reported inadequate oral hygiene practice, e.g. 66.7% (1402) of respondents brushed teeth once a day or less, and 53.6% never visited dentist. Compared with residents who brushed teeth ≥once daily, those who barely or never brushed their teeth were older (68.5 ± 9.4 years), of lower education level, had higher prevalence of lifestyle risk factors and hypertension, had lower waist circumference, visited dentist less frequently, and most of them (82.3%) lived in rural areas. Women tended to have better oral health practice, e.g. 31.2% of respondents who brushed teeth at least twice a day and 35.1% of those brushing once a day were men. Mean ± SD BMI was 24.2 ± 3.3 kg/m^2^ for individuals brushing teeth at least twice a day, comparable to individuals brushing once a day (24.3 ± 3.6 kg/m^2^). Those who barely or never brushed teeth had the least mean BMI (22.7 ± 3.2 kg/m^2^). The highest level of fasting plasma glucose, 2-hour plasma glucose and HbA1c were consistently found in respondents who barely or never brushed their teeth, whilst the lowest level of the three indictors were all found in those brushing teeth at least twice a day (Table 1). These basic characteristics were also described by urban/rural residency in the online appendix (etable).

In urban areas, the mean fasting plasma glucose was 5.12 (95% CI: 5.02–5.23) mmol/L among individuals who brushed teeth at least twice a day, significantly lower than those who brushed teeth once a day (5.59, 95% CI: 5.47–5.72) and those barely brushing teeth (5.98, 5.73–6.22), but no significant difference was found between the latter two groups (Table 2). Similarly among rural residents, mean fasting plasma glucose among individuals brushing at least twice a day (5.31, 5.19–5.44) was significant lower than those with less toothbrushing frequency. Both urban residents who rarely brushed their teeth (8.33, 7.59–9.06) and those brushing once a day (7.53, 7.25–7.82) had significantly higher 2-hour plasma glucose compared with those brushing at least twice a day (7.02, 6.74–7.30); rural residents who rarely or never brushed teeth was 7.80(7.45–8.16) mmol/L, higher than those brushing at least twice a day (6.69, 6.57–7.35). All pairwise comparisons of mean HbA1c among toothbrushing frequency subgroups were found statistically significant for both urban and rural areas, with highest level observed in individuals who rarely or never brushed teeth (urban: 5.64%, 5.49–5.79%; rural: 5.61%, 5.55–5.66%).

In further multivariate models, effects of toothbrushing frequency on fasting plasma glucose, 2-hour plasma glucose and HbA1c were determined (Table 3). On average, urban residents who barely or never brushed their teeth had 0.50 (95% CI: 0.10–0.90) mmol/L higher fasting plasma glucose compared with those brushing teeth at least twice a day, while in rural areas the effect was reduced to 0.26 (0.05–0.48) mmol/L; compared with those brushing teeth at least twice a day, urban residents who brushed teeth once daily was 0.31 (0.14–0.48) mmol/L higher, while was 0.19 (0.01–0.36) mmol/L higher in rural residents. No significant effect of toothbrushing frequency were found against 2-hour plasma glucose, after controlling multiple covariates in the model. With regard to HbA1c, the effect for frequency of rarely or never was 0.26% (0.04–0.47%), and for once a day was 0.12% (0.03–0.22%) in urban areas; in rural areas, the effect was attenuated: 0.20% (0.09–0.31%) for rarely or never and 0.09% (0.004–0.18%) for once a day.

## Discussion

Mean fasting plasma glucose, 2-hour plasma glucose, and HbA1c were higher in individuals who brushed their teeth less frequently. We examined the effects of toothbrushing on fasting plasma glucose, 2-hour plasma glucose, and HbA1c controlling for multiple potential confounding factors. Statistically significant effects were found of toothbrushing on fasting plasma glucose and HbA1c. To the best of our knowledge, this is the first study to quantify effect of a single item self-reported measure of toothbrushing on blood glucose and HbA1c in a large population based survey.

The role oral health plays in the etiology of diabetes has received great attention. Periodontal disease is a prevalent complex chronic subclinical inflammation that the majority of affected people do not notice. It causes a loss of bone support and connective tissue of the teeth^15^. According to a report of World Health Organization, the most sever periodontal disorder affects up to 10–20% of global population, and was considered as the major cause of teeth loss^16^. Despite of the high prevalence, periodontal disease is highly prevented and can be treated to better glycemic control in people with diabetes^17^. Improving oral hygiene is an effective way for the prevention^15^.

The present research is one of the limited studies reporting population-level HbA1c in China. The latest national survey on diabetes in China reported that, in 2010, overall average HbA1c for Chinese adults is 5.8%, and only 4.6% had HbA1c ≥6.5%^2^, the cut-off point defining diabetes according to the American Diabetes Association 2010 criteria^18^. As only individuals without previously diagnosed diabetes were included in the analysis, we found a lower average HbA1c of 5.4% in the study population, with comparable means in rural (5.5%) and urban areas (5.3%).

Relative to brushing teeth at least twice a day, we found that rarely or never brushing teeth is associated, on average, with a rise of 0.50 mmol/L in fasting plasma glucose among urban residents, and a rise of 0.26 among rural residents. These results expanded findings from several cross-sectional epidemiological studies which indicated a correlation between periodontitis and impaired fasting glucose in population without diabetes^8^ and in diabetic patients^9^. Our results on association between toothbrushing frequency and HbA1c level confirmed findings from previous observational studies and clinical trials. For example, the National Health and Nutrition Examination Survey III indicated that higher prevalence of severe periodontitis was found in adults with HbA1c >9.0% compared to individuals without diabetes^7^. An Indian study suggested that HbA1c levels of both individuals without diabetes and with periodontitis reduced significantly 3 months after non-surgical periodontal therapy^19^. Another randomized clinical study reported HbA1c of diabetic patients decreased by 0.86% after 3 months non-surgical periodontal therapy^20^. As the association between toothbrushing and 2-hour blood glucose was not significant, statistical power of the multivariate model was checked. By using power and sample size analysis tool for linear models provided in SAS^21^, we estimated sample size required to reach different level of power. The results showed that the current study provided a statistical power >85% in the multivariate regressions model for 2-hour blood glucose (efigure). Mechanisms that hold back the relationship between toothbrushing and 2-hour blood glucose remain unclear, and additional research is needed to address the reason. However, the association of oral health practice with fasting glucose and HbA1c in a general population without diagnosed diabetes could serve as useful evidence to suggest that good oral hygiene may help reduce fasting glucose blood and HbA1c, even before development of diabetes.

The effects of toothbrushing on blood glucose or HbA1c imply certain epidemiological significance. For example, assuming the fasting blood glucose of a population who never brush teeth follows a normal distribution with a known mean and SD (Table 1), the proportion of raised fasting blood glucose (e.g. ≥7.0 mmol/L) can be easily evaluated by accumulative normal distribution function. If the toothburshing frequency of the population increased to at least twice a day, the average fasting blood glucose would decrease by a certain amount according to the estimated effects (Table 3), resulting in an decrease in the percentage of suspected diabetes in the population. From prevention’s perspective, for a population with poor oral hygiene, a number of diabetes could be avoided if oral hygiene is improved.

Association between oral hygiene practice and diabetes is bidirectional^22^. On one hand, periodontitis could be a potential complication of diabetes. On the other, recent studies suggest that chronic subclinical inflammation intermediates in the pathogenesis of type 2 diabetes^23^,^24^. Increased levels of inflammatory markers, such as C-reactive protein and interleukin-6 are found to be significant risk factors of type 2 diabetes^24^. Periodontal disease may have an increased effect to the inflammatory burden and lead to elevated risk of diabetes. Systemic inflammation, therefore, could explain the underlying mechanism which links oral health and diabetes.

The present study used data from a population-based survey, with a rigorous design. The findings of the analysis, thus, are of good reliance and can be generalized to the general population in the study area. Though the random survey design and big sample size might strengthen our findings, the study still suffered from several limitations. The cross-sectional nature of the study precluded causal inference of study variables. Recall bias and misclassification of socioeconomic characteristics or lifestyle risk factors might play a role in the analysis. Periodontal disease, which mediates the association between toothbrush and diabetes, was not examined in the study due to budget constraint of the survey. Only toothbrushing was studied due to limited information on oral hygiene practices, though it was the most important one. Other oral health behaviors, such as use of dental floss or mouthwash, may need to be examined in further study. The toothbrushing frequency might be over-reported due to embarrassment of reporting poor oral health behavior in the presence of a strange interviewer, and therefore result in narrowed differences between groups and then underestimated effects. However, in large scale population studies use of a single indicator of self-reported toothbrushing could be of great cost effectiveness.

In conclusion, the present study revealed that poor oral practice is associated with increased fasting blood glucose and HbA1c in general population. Establishing good oral health behavioral habits may help prevent diabetes in the general population.

## Methods

### Data source

Data used in this study was extracted from Chongqing Health Behavior and Disease Burden Survey 2012. This survey was a community based random sampling survey aiming to collect information on health related lifestyle behaviors and main diseases affecting health in Chongqing, a southwest municipality of P. R. China. Survey interviews were administrated by qualified field workers. The survey adopted a multistage sampling scheme to reach a representative sample. Nine survey sites (districts/counties) were selected from a total of 38 districts/counties by systematic probability proportional to size (PPS) with sorted urbanization rate (percentage of urban population). Within each selected survey site, 3 townships were chosen using population based PPS method. Then, 2 villages or residential committees were sampled by PPS in each selected township, with one village randomly assigned to group for blood glucose test, and the other assigned to group for lipid test. Finally, 100 households were sampled and within each an adult were sampled, both by simple random sampling. A total of 4485 individuals participated in the survey, representing 29.9 million population in Chongqing. The overall response rate were 83%. There were 2267 respondents in the group for blood glucose test, with a response rate of 84%. Written informed consent was obtained for each respondent before data collection. The ethical review committee of Chongqing Medical University approved the survey protocols. The survey were carried out strictly in accordance with the approved guidelines.

Considering possible reduced blood glucose and HbA1c due to anti-diabetic treatment or lifestyle modification among patients who were aware of their diabetes, we only extracted 2181 observations without previously diagnosed diabetes from the group for blood-glucose test. After excluding 23 individuals who refused to have blood taken and 53 with other missing study variables, there were 2105 individuals remained in the final analysis.

## Measurements

### Blood glucose and HbA1c

Blood samples were required to be collected for every participant after overnight fasting (at least 10 hours). For those in the group for blood-glucose test, a standard 75g glucose solution was given to the participants who reported not being diagnosed diabetes previously by health professionals, and plasma glucose were tested before and at 2 hours after during the oral glucose tolerance test. As we, in this study, only included individuals without diagnosed diabetes, all of them participated in the oral glucose tolerance test. Vacuum blood-collection tubes with anticoagulant sodium fluoride were used to collect and store blood specimens which were centrifuged within 2 hours of collection. Plasma glucose was measured by hexokinase methods within 12 hours at local cooperative health centers or hospitals. Capillary blood samples were also collected from a finger or ear lobe of each participant. All the capillary blood specimens were shipped and stored at 2 °C to 8 °C, and capillary HbA1c were tested within 1 week since collection using high-performance liquid chromatography in central laboratory of the survey. All field workers were trained and only those qualified were allowed to perform blood collection. All laboratorial practices were performed following a standard operating procedure.

### Toothbrushing and other covariates

Questionnaire-based interviews were administrated by trained interviewers to obtain information on demographic characteristics, socio-economic status, diabetes relevant lifestyle risk factors, as well as oral health behaviors. Toothbrushing frequency (≥twice a day, once a day, less than once a day, never) and time since last dental visit (<1 year, 1–2 years, 3–4 years, >5 years, never) were assessed. A composite indicator was constructed of number of five diabetes related lifestyle risk factors, including current smoking, excessive alcohol drinking, physical inactivity, high red meat intake and low fruit and vegetable intake. Current smoking was defined as self-reported tobacco use every day or on some days at the time when the survey was carried out. Consumption of alcohol, vegetable and fruit, and red meat were measured using a food frequency questionnaire. According to the Dietary Guidelines for Chinese Residents^25^, over drinking was defined as a pure alcohol intake of ≥15 g/day for women and ≥25 g/day for men. Consuming less than 400 g/day of fruit and vegetables was considered as insufficient^26^. A diet high in red meat was defined as consuming ≥100 g/day of beef, pork, or lamb^27^. Physical inactivity was defined as less than 150 minutes of moderate-intensity activity per week, or equivalent^28^. Body mass index (BMI) was computed using an objectively measured height and weight of each participant. Hypertension was defined as systolic blood pressure ≥140 mm Hg, diastolic blood pressure ≥90 mm Hg, or had been diagnosed by a health professional.

### Statistical analysis

Characteristics of study population were explored by toothbrushing frequency and urban/rural residency. Means and 95% confidence intervals (95% CI) of fasting plasma glucose, 2-hour plasma glucose after drinking glucose solution, and HbA1c were determined according to toothbrushing frequency. Between groups means were compared and tested by analysis of variance. Probability of multiple comparisons were adjusted using Scheffé’s method. In further multi-variable analysis, we used general linear models to evaluate the effects of toothbrushing frequency on level of fasting plasma glucose, 2-hour plasma glucose and HbA1c, controlling for age, sex, marital status (3 levels: Single, Married or cohabiting, Separated/divorced/widowed/others), education attainment (5 levels: Illiterate, Primary school, Junior high school, Senior high school, College graduate or above), household income per capita (4 levels in quartiles), number of lifestyle risk factors (4 levels: 0, 1, 2, ≥3), hypertension status (2 levels: with, without), waist circumference, and time since last dental visit (4 levels: <1 year, 1–2 years, ≥3 years, never). Considering marked discrepancy of oral health and diabetes prevalence between Chinese urban and rural residents, analyses were stratified by residential location^1^,^2^,^5^. All analysis were performed in SAS 9.4. Statistical significance was at the 0.05 level.

## Additional Information

**How to cite this article**: Su, L. *et al*. Toothbrushing, Blood Glucose and HbA1c: Findings from a Random Survey in Chinese Population. *Sci. Rep.* **6**, 28824; doi: 10.1038/srep28824 (2016).

## Supplementary Material

Supplementary File 1

## Figures and Tables

**Table 1:** Characteristics of study population in relation to frequency of toothbrushing.

	Frequency of toothbrushing			Total
	At least twice a day	Once a day	Rarely or never	
Participants–no.	703	1118	284	2105
Mean age (SD)–years	56.2 (14.2)	59.6(11.8)	68.5(9.4)	59.6(12.9)
Male sex–%	31.2	35.1	50.5	35.8
Living in couples–%	20.5	15.7	23.8	18.4
No education–%	22.6	40.4	68.2	38.2
Without lifestyle risks–%	32.4	30.4	25.1	30.4
Employed–%	60.2	69.7	79.2	67.8
Living in urban areas–%	69.6	45.6	17.7	49.9
Never had dental visit–%	47.1	54.1	67.5	53.6
Hypertension–%	31.9	36.4	39.9	35.4
Waist circumference (SD)–mm	82.5(9.3)	83.2(9.7)	80.7 (9.3)	82.6 (9.6)
BMI (SD)–kg/m2	24.2(3.3)	24.3(3.6)	22.7(3.2)	24.1(3.5)
Fasting plasma glucose (SD)–mmol/L	5.2(1.2)	5.6(1.3)	5.7(0.8)	5.5(1.2)
2-hour plasma glucose (SD)–mmol/L	7.0(3.1)	7.4(3.1)	7.9(3.1)	7.4(3.1)
Hemoglobin A1c (SD)–%	5.2(0.7)	5.4(0.7)	5.6(0.5)	5.4(0.6)

**Table 2:** Means of fasting plasma glucose, 2-hour plasma glucose, and hemoglobin A1c by toothbrushing frequency*.

Toothbrushing frequency	Fasting plasma glucose (mmol/L)		2-hour plasma glucose (mmol/L)		Hemoglobin A1c(%)	
	Urban	Rural	Urban	Rural	Urban	Rural
At least twice a day	5.12 (5.02, 5.23)^a^	5.31(5.19, 5.44)^a^	7.02(6.74, 7.30)^a^	6.69(6.57, 7.35)	5.17(5.11, 5.23)^a^	5.34(5.26, 5.42)^a^
Once a day	5.59 (5.47, 5.72)	5.54(5.45, 5.64)	7.53(7.25, 7.82)	7.34(7.11, 7.58)	5.40(5.33, 5.46)^b^	5.46(5.41, 5.50)^b^
Rarely or never	5.98(5.73, 6.22)^c^	5.66(5.56, 5.76)^c^	8.33(7.59, 9.06)^c^	7.80(7.45, 8.16)^c^	5.64(5.49, 5.79)^c^	5.61(5.55, 5.66)^c^

^a^Difference was significant at the 0.05 level between at least twice a day and once a day.

^b^Difference was significant at the 0.05 level between once a day and rarely or never.

^c^Difference was significant at the 0.05 level between at least twice a day and rarely or never.

**Table 3:** Coefficients in general linear models to estimate the effects of toothbrushing frequency on fasting plasma glucose, 2-hour plasma glucose, and hemoglobin A1c*.

	Toothbrushing frequency	Fasting plasma glucose (mmol/L)		2-hour plasma glucose (mmol/L)		Hemoglobin A1c (%)	
		Coefficient (%)	p value	Coefficient (%)	p value	Coefficient (%)	p value
Models for urban residents	At least twice a day	0		0		0	
	Once a day	0.31(0.14, 0.48)	<0.01	0.19(−0.23, 0.60)	0.37	0.12(0.03, 0.22)	<0.01
	Rarely or never	0.50(0.10, 0.90)	0.02	0.70(−0.27, 1.66)	0.16	0.26(0.04, 0.47)	0.02
Models for rural residents	At least twice a day	0		0		0	
	Once a day	0.19(0.01, 0.36)	0.03	0.34(−0.12, 0.79)	0.15	0.09(0.00, 0.18)	0.04
	Rarely or never	0.26(0.05, 0.48)	0.02	0.44(−0.14, 1.02)	0.14	0.20(0.09, 0.31)	<0.01

^*^Models were adjusted for age, sex, marital status, education, household income per capita, lifestyle risk factors, BMI, time since last dental visit, hypertension and waist circumference.
