# Supplementary material for: Toothbrushing, Blood Glucose and HbA1c: Findings from a Random Survey in Chinese Population
Source: Sci Rep. 2016 Jul 7;6:28824. doi: 10.1038/srep28824 (PMC4935949; doi:10.1038/srep28824)
Supplement: Supplementary File 1 [file srep28824-s1.doc]

Article title: Toothbrushing, Blood Glucose and HbA1c: Findings from a Random Survey in Chinese Population

Author list: Lingyu Su, Wenzhao Liu, Bingwu Xie, Lei Dou, Jun Sun, Wenjuan Wan, Xiaoming Fu, Guangyue Li, Jiao Huang, Ling Xu

Etable Characteristics of study population in relation to frequency of toothbrushing by urban/rural residency.

|  | Twice a day | |  | Once a day | |  | Rarely or never | |  | Total | |
| --- | --- | --- | --- | --- | --- | --- | --- | --- | --- | --- | --- |
|  | Urban | Rural |  | Urban | Rural |  | Urban | Rural |  | Urban | Rural |
| Participants - no. | 489 | 214 |  | 510 | 608 |  | 51 | 233 |  | 1050 | 1055 |
| Mean age (SD) - years | 55.8(13.9) | 57.1(15) |  | 59.2(11.4) | 59.9(12.1) |  | 67.8(11.3) | 68.7(8.9) |  | 58(12.9) | 61.2(12.8) |
| Female sex - % | 30.7 | 32.2 |  | 32 | 37.7 |  | 42 | 52.4 |  | 31.8 | 39.8 |
| Living in couples - % | 19.8 | 220 |  | 14.9 | 16.3 |  | 30 | 22.4 |  | 17.9 | 18.8 |
| No education - % | 13.3 | 43.9 |  | 31 | 48.2 |  | 62 | 69.5 |  | 24.2 | 52 |
| Without lifestyle risks - % | 32.1 | 33.2 |  | 30.2 | 30.6 |  | 20 | 26.2 |  | 30.6 | 30.1 |
| Employed - % | 50.1 | 83.2 |  | 52.7 | 84 |  | 58 | 83.7 |  | 51.7 | 83.8 |
| Never had dental visit - % | 47 | 47.2 |  | 49.6 | 57.9 |  | 72 | 66.5 |  | 49.5 | 57.6 |
| Hypertension - % | 39.9 | 46.4 |  | 47.9 | 45.2 |  | 65.3 | 56.9 |  | 45 | 48.1 |
| Waist circumference (SD) - mm | 82.5(9.4) | 82.5(9.1) |  | 84.8(9.5) | 81.8(9.7) |  | 80.4(9.4) | 80.8(9.3) |  | 83.6(9.5) | 81.7(9.5) |
| BMI (SD) - kg/m2 | 24.4(3.3) | 23.8(3.2) |  | 25.0(3.4) | 23.8(3.7) |  | 22.9(3.3) | 22.7(3.2) |  | 24.6(3.4) | 23.6(3.5) |
| Fasting plasma glucose (SD) - mmol/L | 5.1(1.2) | 5.3(0.9) |  | 5.6(1.4) | 5.5(1.2) |  | 6.0(0.9) | 5.7(0.8) |  | 5.4(1.3) | 5.5(1.1) |
| 2-hour plasma glucose (SD) - mmol/L | 7.0(3.1) | 7.0(2.9) |  | 7.5(3.3) | 7.3(3) |  | 8.3(2.6) | 7.8(2.7) |  | 7.3(3.2) | 7.4(2.9) |
| Hemoglobin A1c (SD) - % | 5.2(0.7) | 5.3(0.6) |  | 5.4(0.7) | 5.5(0.6) |  | 5.6(0.5) | 5.6(0.4) |  | 5.3(0.7) | 5.5(0.5) |


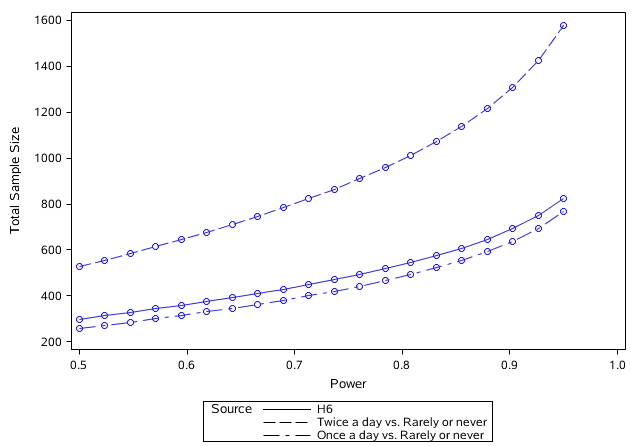


Efigure Sample size required with different statistical power in multivariate regression model against blood glucose at 2 hour*

*we assumed that never brushing group has a higher mean, and by defining two-sided alpha as 0.025, the number of covariates as 10, standard deviation for 2-hour glucose as 3.1 (table 1), correlation for 2-hour glucose and BMI (the most important continuous covariate) as 0.35 (estimated by the current data).

H6 in the figure is the variable name of toothbrushing frequency.
